# A design of a targeted puncture trajectory applied to unilateral extrapedicular percutaneous vertebroplasty

**DOI:** 10.1186/s12891-023-06387-w

**Published:** 2023-04-05

**Authors:** Tao Zhang, Yinghu Deng

**Affiliations:** Department of Spine Surgery, Tongling People’s Hospital, Tongling, 244000 Anhui China

**Keywords:** Compression, Fractures, Vertebroplasty, Bone cements

## Abstract

**Objective:**

In this study, we introduced a design of a targeted puncture trajectory applied to unilateral extrapedicular percutaneous vertebroplasty.

**Methods:**

62 individuals with osteoporotic vertebral compression fractures (OVCF) were included in this research at the Tongling People’s Hospital, from January 2019 to December 2020. Percutaneous Vertebroplasty (PVP) was performed on all patients using a targeted unilateral extrapedicular puncture technique guided by G-arm fluoroscopy. The operating time, volume and dispersion of bone cement, and cement leak were all evaluated. The oswestry disability index(ODI) and the visual analog scale (VAS) were used to assess pain relief and quality of life (QOL).

**Results:**

The targeted puncture trajectory for unilateral extrapedicular PVP was used to successfully treat a total of 62 fractured vertebrae without any apparent clinical issues. In comparison to their equivalent preoperative values, the VAS and ODI values after surgery were considerably lower (*P < 0.01*). The bone cement not only could be across the midline of the targeted vertebrae but also appeared in both the bilateral pedicle and the center projection region on the anteroposterior X-ray film, according to radiologic results in all injured vertebrae. There were 3 cases of leakage at the anterior border of the vertebral body and 2 cases of leakage into the intervertebral region without significant clinical manifestations. Furthermore, no bone cement leaked into the vessels or spinal canal.

**Conclusion:**

The design of the targeted puncture trajectory used in unilateral extrapedicular PVP not only ensures that the bone cement injector transcends the midline of the vertebral body, but it also improves the accuracy of the injector arriving at the contralateral pedicle projection area. As a result, this approach can increase well-distributed bone cement diffusion while preventing cement leakage into the spinal canal.

## Introduction

The prevalence of osteoporotic vertebral compression fracture (OVCF) is rising, particularly in the thoracolumbar vertebral body, as the population ages. The elderly patient’s ability to care for themselves and their quality of life has been severely impacted by pain [1]. It has compelled surgeons to pay closer attention to the physical and mental well-being of older OVCF patients. Percutaneous vertebroplasty (PVP), an advanced minimally invasive therapy, is routinely performed in numerous countries to treat the discomfort caused by OVCF, vertebral hemangiomas, and vertebral tumors [2–4].

There are mainly two methods for PVP: one is a unilateral puncture, and the other is bilateral. According to reports, during the PVP procedure, the direction and location of the bone cement injector directly affect the volume of bone cement and the surgery’s safety [5–7].

The conventional unilateral puncture procedure sometimes fails when the cement injector cannot traverse the midline of the vertebral body and the bone cement cannot distribute in the contralateral pedicle projection region on the anteroposterior X-ray film. While consistent bone cement diffusion inside the vertebra is necessary to ensure surgical efficacy [8]. In this study, we designed a targeted puncture method for unilateral extrapedicular percutaneous vertebroplasty and demonstrated its efficiency and safety.

## Materials and methods

This is a retrospective study. From January 2019 to December 2020, 62 patients with single-segment OVCF who received PVP in our institution were evaluated. All patients who were enrolled signed written forms of informed consent. Ethical approval was provided by the ethical committee of Tongling People’s Hospital (No.20,181,210). All individuals were followed up for a year. For these individuals, the adoption procedure included a typical outpatient clinic appointment, a follow-up visit, or a phone call.

### Inclusion criteria

1. Acute or chronic single segmental OVCF (T11-L1).
2. A hypointense signal was discovered in a T1-weighted magnetic resonance imaging (MRI) image. A hyperintense signal was visible on an MRI picture of fat suppression.
3. Patients above the age of 60 were diagnosed with OVCF caused by minor or moderate trauma.
4. Acute or chronic back pain with VAS was 5 scores or more.
5. BMD T-score was minus 2.5 SD or below.

### Exclusion criteria

1. Pathologic fractures except osteoporotic fractures (e.g., cancer with metastasis to the vertebrae, suppurative spondylitis).
2. Neurologic deficit.
3. Vertebral canal stenosis or compression of the spinal cord.
4. Untreatable bleeding disorders.
5. Significant comorbidity in the heart, lungs, or kidneys that makes surgery impossible.

### The design for targeted puncture trajectory

**Fig. 1:**
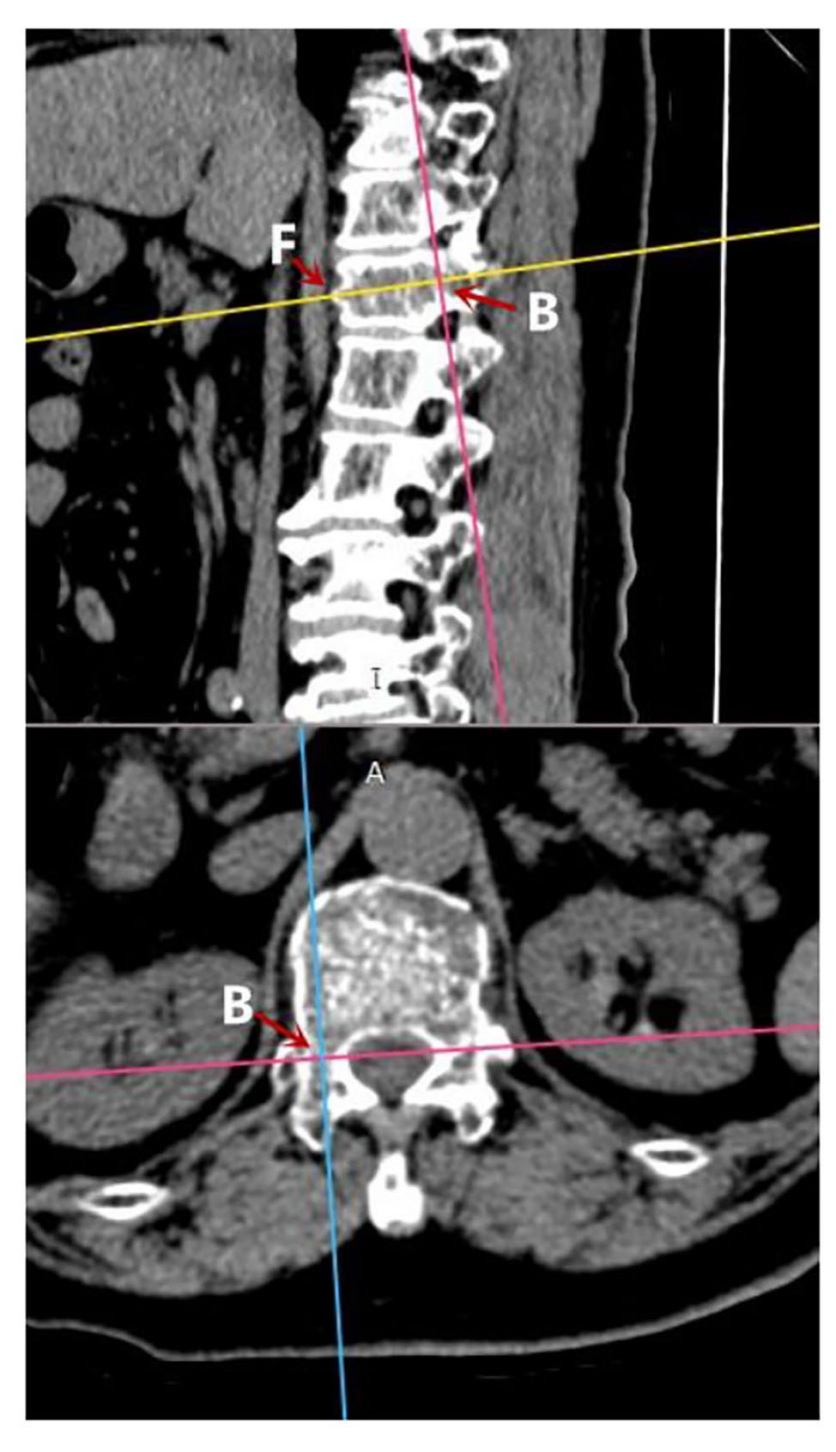
On 3D-CT images of the fractured vertebra, the puncture-side pedicle’s center is designated as point B, and the midpoint of the sagittal anterior edge is point F

**Fig. 2:**
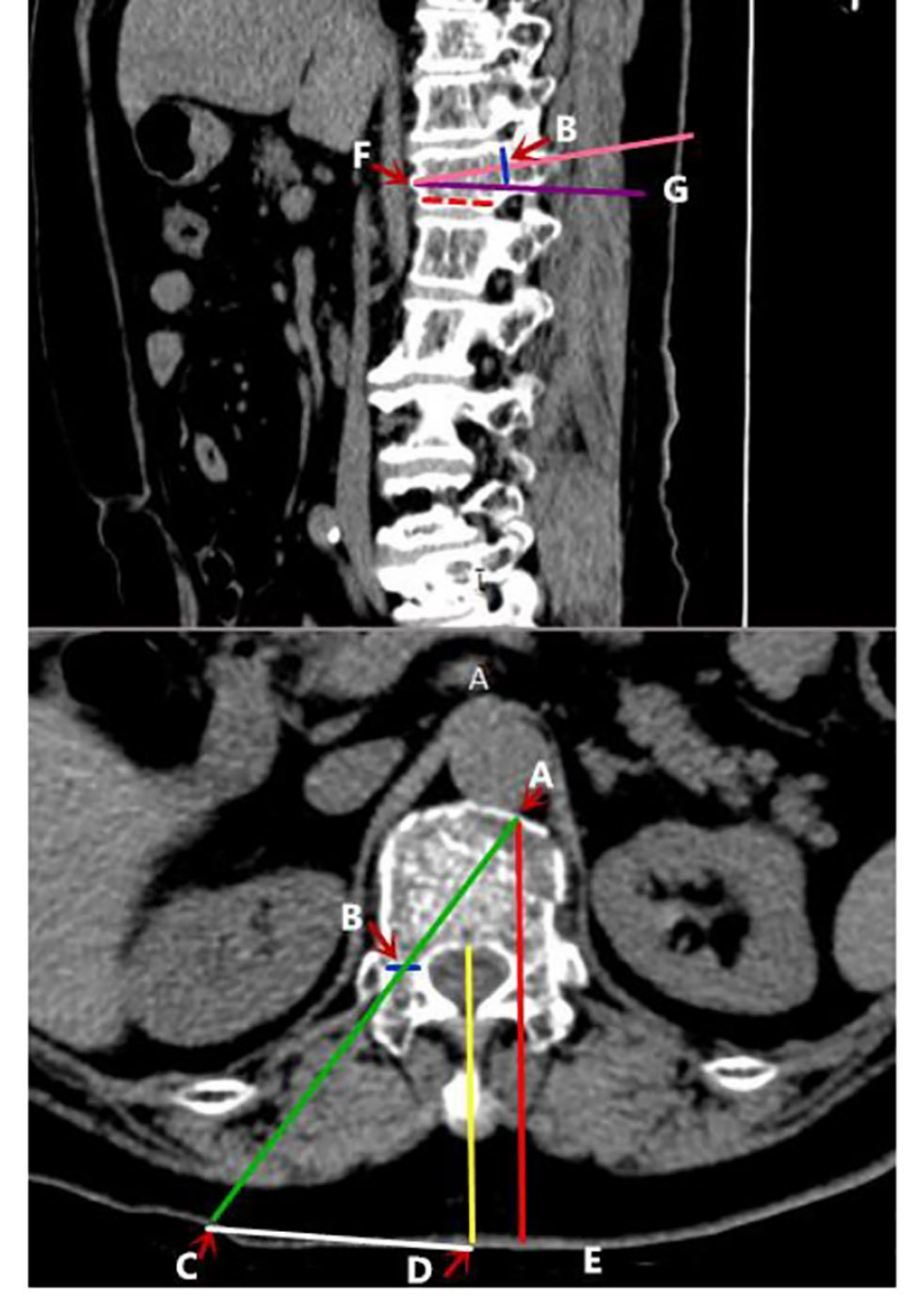
The yellow line is the median sagittal line, and the red line is the tangent line that runs parallel to the yellow line through the inner edge of the contralateral pedicle. The intersection point by intersecting the red line with the anterior border of the fractured vertebra is point A. Point B is the puncture-side pedicle’s center; point C is the puncture site; the distance CD should be measured before surgery; ∠CAE is the abduction angle. The purple line runs parallel to the fractured vertebra’s inferior border; ∠BFG is the sagittal inclination angle

After CT scanning, we imported CT images in DICOM format into PACS(Picture Archiving and Communication System). First of all, the center of the puncture-side pedicle ( point B) and the midpoint of the sagittal anterior edge (point F) were tagged on 3-dimensional computed tomography(3D-CT) images of the fractured vertebra(Fig. 1). Then the puncture trajectory was designed as follows (Fig. 2): The yellow line is the median sagittal line, and the red line is the tangent line that runs parallel to the yellow line through the inner edge of the contralateral pedicle. Point A is the intersection point by having the red line intersect the anterior edge of the fractured vertebra. Point C is the puncture site; the distance CD should be measured before surgery; ∠CAE is the abduction angle. The purple line runs parallel to the fractured vertebra’s inferior border; ∠BFG is the sagittal inclination angle.

### Surgical treatment procedures

**Fig. 3:**
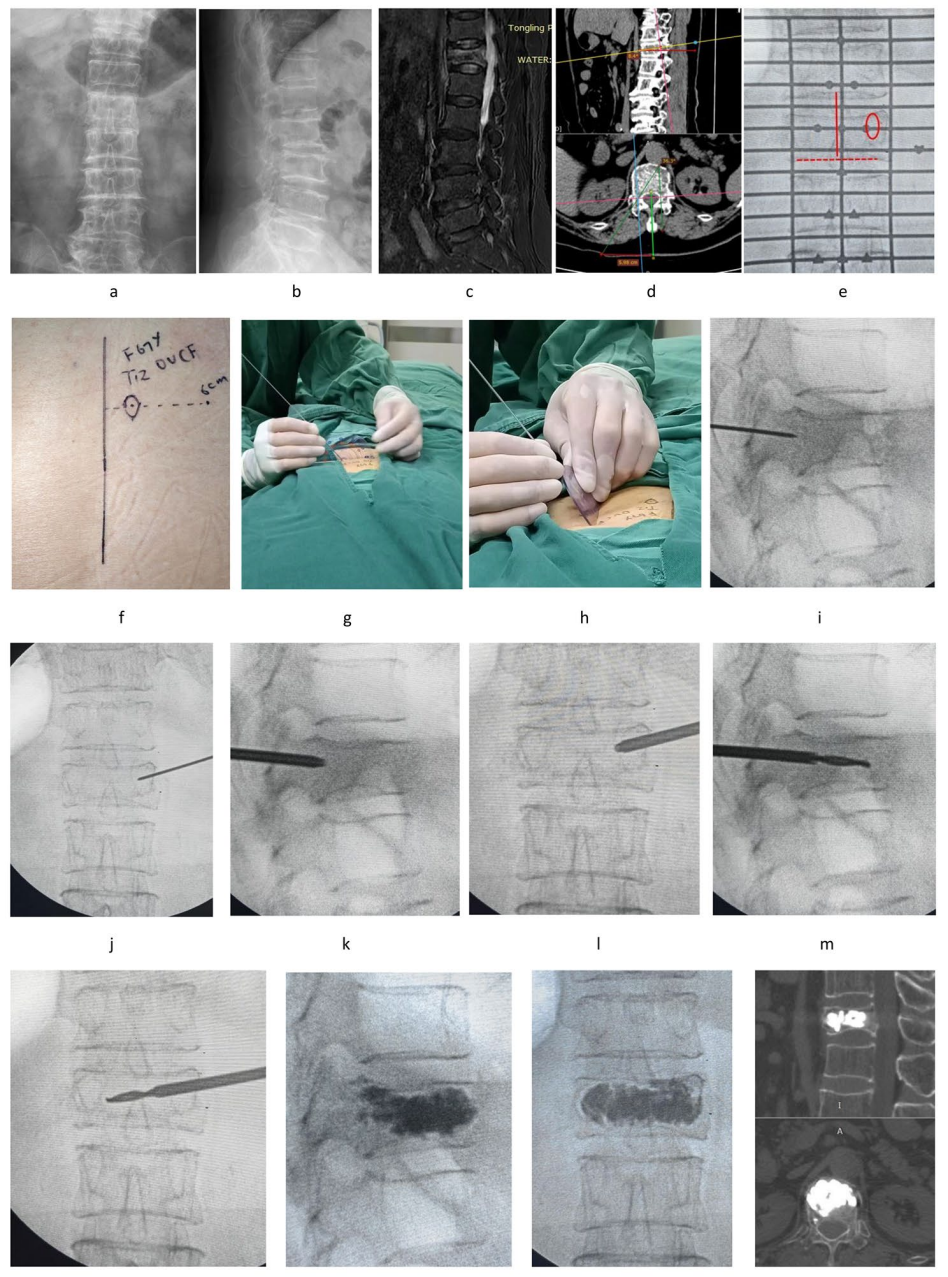
A 67-year-old female individual was diagnosed with OVCF at T12. a,b: Anteroposterior and lateral X-ray films before operation. c: A hyperintense intensity in the fractured vertebra was shown on the fat-suppressed MRI image before surgery. d: The puncture trajectory was designed through PACS. e, f: The puncture-side pedicle projection and the midline of the spine on the patient’s back skin were tagged. The puncture site was then confirmed according to Fig. 3d. Besides, the fractured vertebral inferior border (the red dotted line) does not have a double-layer projection. g, h: The abduction angle and sagittal inclination angle were adjusted by a protractor. i-n: The piercing process was shown. o-q: In the X-ray and 3D-CT pictures following surgery, the bone cement was effectively diffused in the fractured vertebra

Under general anesthesia, the same spinal surgeon conducted all procedures. On begin, patients were positioned prone on the operation table, with a cushion underneath the chest and hips to free up space in the abdomen. The G-shaped arm X-ray fluoroscopy was used to target the fractured vertebra. The perspective position was adjusted on both sides to equalize the distance of the bilateral pedicle’s projection from the spinous process. Additionally, the fractured vertebral inferior border does not have a double-layer projection. At this step, the puncture-side (usually the right side) pedicle projection on the patient’s back skin should be tagged, and its center point should be marked. Subsequently, draw a vertical line from the center point to the middle line of the spine. The puncture site, which was located at a certain distance (distance CD mentioned in Fig. 2) beside the apical spinous, was on the extension cord of the vertical line. Then, a puncture needle was inserted into the skin from the puncture point as measured above. The preoperatively measured abduction angle and sagittal inclination angle were adjusted repeatedly and lightly by a protractor. The needle nearly reached the vertebra’s posterior border projection when it was placed at the medial margin of the puncture-side pedicle projection. At this time, make a 4–5 mm longitudinal (or horizontal) incision in the skin at the puncture site. The fractured vertebrae were then punctured along the trajectory as mentioned above utilizing a Vertebroplasty system (Suzhou AND Co., Ltd., China). The puncture needle was then withdrawn, and a bone drill was inserted along the needle route. Then, the drill tip reached the anterior middle region of the vertebra, it was approximately at the inner border of the contralateral pedicle projection. Following the removal of the drill, a bone cement injector was placed along the drill channel. Lastly, the cement was injected while being monitored by a G-arm fluoroscopy. ( A typical case was shown in Fig. 3)

### Assessments of parameters

Clinical and radiological data were included as evaluation indicators. The relevant clinical assessment data were primarily collected: operation time, bone cement volume, visual analog scale (VAS) and oswestry disability index(ODI) scores at each particular time (preoperation, one day after surgery, and one year after surgery), intraoperative complications, and neurologic issues after surgery. As a radiographic assessment index, we mainly focused on bone cement’s diffusion in the fractured vertebrae.

### Data Analysis

For data analysis, we utilized SPSS 22.0 software. The measurement data was represented as mean ± standard deviation(‾*X±s*). To detect differences in VAS and ODI scores between preoperative and postoperative periods, the one-way ANOVA was used. The P value < 0.05 was considered statistically significant.

## Results

All of the 62 patients’ baseline characteristics and fractured vertebral data were shown in Table 1.

**Table 1:** Baseline characteristics and fractured vertebral data

Gender		Age	Fractured vertebral			BMD T-score	BMI
male	female	(years)	T11	T12	L1	3.32 ± 0.32	23.27 ± 2.67
10	52	70.64 ± 7.04	5	35	22		

BMD, bone mineral density. BMI,Body mass(kg)/Height(cm)^2^

Intraoperatively, the bone cement injector successfully crossed the midline of the vertebrae and reached the inner border of the contralateral pedicle projection area on the anteroposterior X-ray film. All fractured vertebrae had well-distributed bone cement. The single vertebra’s average injected bone cement volume was 5.51 ± 0.48ml. The surgery lasted between 20 and 45 min, with a mean time of 29.21 ± 7.30 min. There were no intraoperative issues (e.g., pulmonary embolism, bone cement allergy) and no neurologic sequelae following surgery in any of the patients. Without significant clinical manifestations, there were 3 cases of leakage at the anterior border of the vertebral body and 2 cases of leakage into the intervertebral space. Besides, there was no bone cement leak into the vessels or spinal canal.

All patients received one year of follow-up. The VAS scores were as follows: 6.14 ± 0.53 before surgery, 1.77 ± 0.71 on the first day after surgery, and 0.91 ± 0.27 at one year after surgery. The ODI was 62.41 ± 4.76 before surgery, 25.16 ± 4.33 on the first day after surgery, and 17.82 ± 4.67 at one year after surgery. The VAS scores and ODI at the first postoperative day were significantly lower than the preoperative but considerably higher than the last follow-up(*P < 0.01*)(Table 2).

**Table 2:** VAS and ODI score variations between the pre- and postoperative periods

	Preop	the first day after surgery	one year after surgery	*P1*	*P2*
VAS	6.14 ± 0.53	1.77 ± 0.71	0.91 ± 0.27	< 0.01	< 0.01
ODI(%)	62.41 ± 4.76	25.16 ± 4.33	17.82 ± 4.67	< 0.01	< 0.01

n = 62; P1, preoperative vs. the first day after surgery; P2, the first day after surgery vs. one year after surgery; n,total number of patients

## Discussion

With advances in the puncture technique, unilateral puncture has become more commonly employed to treat PVP [9, 10]. Several studies have found that unilateral puncture has the same clinical outcomes as bilateral puncture [11, 12]. Unfortunately, we intraoperatively found that inadequate filling of bone cement in the contralateral pedicle projection area happens often in conventional unilateral puncture PVP and that as the cement thickness increases, the dispersion in the contralateral pedicle projection region worsens.

As is well known, a crucial requirement for the clinical outcome of surgery is the symmetric diffusion of cement in the fractured vertebral body. He et al. [8] proved that cement distribution in the vertebral body is a crucial element in determining long-term effects following surgery. They claimed that the “H”-shape distribution of bone cement in vertebrae was superior to the “O”-shape distribution. The “H” distribution indicates that, in the vertebra, the bone cement is filled in both the bilateral pedicle and the center projection region on the anteroposterior X-ray film. It is not difficult to understand that the “H”-shaped cement form in the vertebral body makes its force more similar to the “platform” support, while the “O”-shaped cement distribution makes its force more inclined to the “point” support. The “platform” support can easily reduce the movement between bone trabeculae in the vertebral body. Simultaneously, micromotion between bone trabeculae has been considered one of the causes of residual pain after vertebroplasty [13, 14]. Therefore, to decrease the trabeculae’s micromovement in the fractured vertebra, we should make the bone cement in the vertebra as equally spread as possible intraoperatively. Whereas, the typical unilateral puncture procedure is more likely to result in an “O”-shaped distribution rather than an “H”-shaped distribution [8]. To avoid “O”-shaped bone cement dispersion and overcome the condition that cement cannot distribute in the bilateral pedicle projection region during a unilateral puncture procedure, we aimed the target of the bone cement injector at the intersection point by intersecting the tangent line that goes through the inner margin of the contralateral pedicle with the anterior edge of the fractured vertebra during preoperative measurement planning. Based on this arrangement, bone cement could initially diffuse into the contralateral pedicle projection area.

Due to the limited pedicle width in the thoracolumbar vertebrae, during the PVP procedure, we must ensure the spinal canal is not injured while inserting the working cannula into the fractured vertebral body. According to Lien et al. [15], the pedicle width in T11-L1 is 7.4, 7.4, and 6.4 mm, respectively. In PVP, the working cannula diameter is typically 4.0 mm. In this research, we used PACS software to evaluate CT images of fractured vertebrae before surgery thoroughly. We determine three key points in the 3D-CT images of the fractured vertebra during the design process of the puncture trajectory before surgery: (1) The first is the center of the puncture-side pedicle. Here, it needs to be noted that, although the puncture-side in this study was always on the right, it practically should be determined based on the patient’s characteristics. (2) The second is obtained by intersecting the tangent line that goes through the inner margin of the contralateral pedicle with the anterior edge of the fractured vertebra. (3) The third is the midpoint of the sagittal anterior edge. This design offers three advantages owing to the three key points: (1) The puncture route guarantees that the working cannula can enter the vertebral body through the middle point of the puncture-side pedicle, effectively preserving the inner wall of the puncture-side pedicle from injury. (2) Intraoperatively, the bone cement injector not only can exceed the vertebral midline, but it also improves the injector’s accuracy in reaching the contralateral pedicle projection area. As a result of this, bone cement can penetrate the midline of the fractured vertebra and distribute effectively. (3) Due to a precise design before surgery based on these three key points using PACS software, the operational procedure was uncomplicated and controlled.

This study has several limitations. Firstly, it covered a limited number of patients and did not evaluate the adjacent vertebrae as a parameter. Secondly, this design is limited to the T11, T12, and L1 vertebrae. Thirdly, the preoperative CT measurement is still different from the actual G-arm fluoroscopy during the real surgical procedure, which requires us to repeatedly and lightly confirm the trajectory of the puncture direction. To justify and generalize our design, we would additionally conduct a control study and enroll a large number of samples.

## Conclusion

In summary, during a PVP surgery, the design of the puncture trajectory employed in unilateral extrapedicular puncture not only assures that the bone cement injector crosses the midline of the vertebrae, but also increases the injector’s accuracy in reaching the contralateral pedicle projection region. As a result, this approach can improve well-distributed bone cement diffusion in the fractured vertebra while avoiding cement leakage into the spinal canal.

## Data Availability

The datasets used and/or analyzed during the current study are available from the corresponding author on reasonable request.
